# HIV-1 envelope glycan modifications that permit neutralization by germline-reverted VRC01-class broadly neutralizing antibodies

**DOI:** 10.1371/journal.ppat.1007431

**Published:** 2018-11-05

**Authors:** Celia C. LaBranche, Andrew T. McGuire, Matthew D. Gray, Shay Behrens, Tongqing Zhou, Quentin J. Sattentau, James Peacock, Amanda Eaton, Kelli Greene, Hongmei Gao, Haili Tang, Lautaro G. Perez, Kevin O. Saunders, John R. Mascola, Barton F. Haynes, Leonidas Stamatatos, David C. Montefiori

**Affiliations:** 1 Department of Surgery, Duke University Medical Center, Durham, NC, United States of America; 2 Fred Hutchinson Cancer Research Center, Department of Global Health, Seattle, WA, United States of America; 3 Vaccine Research Center, National Institute of Allergy and Infectious Diseases, National Institutes of Health, Bethesda, Maryland, United States of America; 4 The Sir William Dunn School of Pathology, University of Oxford, South Parks Road, Oxford, United Kingdom; 5 Duke University School of Medicine, Departments of Medicine and Immunology, Duke Human Vaccine Institute, Durham, NC, United States of America; 6 University of Washington, Department of Global Health, Seattle, Washington, United States of America; Emory University, UNITED STATES

## Abstract

Broadly neutralizing antibody (bnAb) induction is a high priority for effective HIV-1 vaccination. VRC01-class bnAbs that target the CD4 binding site (CD4bs) of trimeric HIV-1 envelope (Env) glycoprotein spikes are particularly attractive to elicit because of their extraordinary breadth and potency of neutralization *in vitro* and their ability to protect against infection in animal models. Glycans bordering the CD4bs impede the binding of germline-reverted forms of VRC01-class bnAbs and therefore constitute a barrier to early events in initiating the correct antibody lineages. Deleting a subset of these glycans permits Env antigen binding but not virus neutralization, suggesting that additional barriers impede germline-reverted VRC01-class antibody binding to functional Env trimers. We investigated the requirements for functional Env trimer engagement of VRC01-class naïve B cell receptors by using virus neutralization and germline-reverted antibodies as surrogates for the interaction. Targeted deletion of a subset of N-glycans bordering the CD4bs, combined with Man_5_ enrichment of remaining N-linked glycans that are otherwise processed into larger complex-type glycans, rendered HIV-1 426c Env-pseudotyped virus (subtype C, transmitted/founder) highly susceptible to neutralization by near germline forms of VRC01-class bnAbs. Neither glycan modification alone rendered the virus susceptible to neutralization. The potency of neutralization in some cases rivaled the potency of mature VRC01 against wildtype viruses. Neutralization by the germline-reverted antibodies was abrogated by the known VRC01 resistance mutation, D279K. These findings improve our understanding of the restrictions imposed by glycans in eliciting VRC01-class bnAbs and enable a neutralization-based strategy to monitor vaccine-elicited early precursors of this class of bnAbs.

## Author summary

Activation of appropriate naïve B cells is a critical initial step in the elicitation of broadly neutralizing antibodies (bnAbs) by HIV-1 vaccines. Germline-reverted forms of bnAbs partially mimic naïve B cell receptors, making them useful for designing and identifying immunogens that can initiate early stages of bnAb development. Here we identify a combination of glycan-modifications on the HIV-1 envelope glycoproteins that preserve native structure and facilitate interactions with germline-reverted forms of the VRC01-class of bnAbs. These modifications included the complete removal of certain N-glycans, combined with Man_5_-enrichment of remaining N-glycans that otherwise are processed into larger complex-type glycans. HIV-1 Env-pseudotyped viruses modified in this way were highly susceptible to neutralization by germline-reverted forms of several VRC01-class bnAbs, and this neutralization could be blocked by a known VRC01 resistance mutation. These findings provide new insights for the design and testing of novel immunogens that aim to elicit VRC01-like bnAbs.

## Introduction

The CD4-binding site (CD4bs) of HIV-1 envelope glycoproteins (Env) is essential for virus entry [1] and is susceptible to some of the most potent broadly neutralizing antibodies (bnAbs) described to date, neutralizing up to 98% of circulating strains [2–10]. These bnAbs also prevent SHIV infection in nonhuman primates [11–16] and produce transient reductions in plasma viremia in infected humans [17, 18] and macaques [19, 20]. Such features make CD4bs bnAbs highly attractive for vaccine development. Unfortunately, although the human immune system is clearly capable of making these antibodies in the setting of chronic HIV-1 infection, all efforts to elicit them with vaccines in non-human primates and humans have failed [21].

A major roadblock is the high level of somatic hypermutation required to bind an epitope that is conformationally masked and sterically occluded by surrounding glycans [7, 9, 10, 22, 23]. Mature CD4bs bnAbs resemble CD4 in their mode of binding and contact the CD4-binding loop while avoiding or accommodating potential clashes with loop D and the fifth variable (V5) regions of gp120, often contacting both of these latter regions [2, 8, 22, 24]. Few immunoglobulin gene families appear to give rise to CD4bs bnAbs, most notably VH1-2 and the closely related VH1-46, both of which are utilized by the most potent CD4bs bnAbs (e.g., VRC01, 3BNC117, N6, CH235.12). Binding of these bnAbs is mediated by the heavy and light chains and is dominated by the heavy-chain second complementarity determining region (CDRH2) when either VH1-2 or VH1-46 are utilized [2, 5, 10]. Other CD4bs bnAbs (e.g., CH103, VRC13, VRC16 and HJ16) make use of multiple additional VH gene families, and their binding involves a CDRH3-dominated mode of recognition [6, 10].

Most current immunogens fail to bind germline-reverted forms of CD4bs bnAbs [7, 9, 22, 25–29] and therefore are unlikely to engage cognate naïve B cell receptors (BCRs). Weak germline binding has been detected against autologous Envs but it is not clear that this weak binding will provide an adequate stimulus to initiate bnAb development [30, 31]. Relationships between antibody structure and function are serving as a basis to reverse-engineer improved germline-targeting immunogens for the VRC01 class of CD4bs bnAbs. Notably, germline-reverted forms of these bnAbs are less positively charged [32] and their CDRH3 might play a more dominant role [33] than the mature bnAbs; both of these features could potentially influence interactions with complex-type glycans.

Germline binding has been detected by introducing Env mutations that selectively remove glycans in the vicinity of the CD4bs that are predicted to clash with germline forms of the bnAbs. Targeted removal of three glycans from clade C strain 426c gp140, one at N276 in loop D that contacts the light chains of VRC01 and NIH45-46 [22, 34], and two at N460 and N463 in V5 that modulate VRC01 sensitivity [35], permit nanomolar avid binding of germline-reverted forms of VRC01 and NIH45-46 [27]. These mutations also permit activation of B cells expressing germline-reverted BCRs of VRC01 and NIH45-46 *in vitro* [27]. Further modifications to the 426c Env, including the removal of the first, second and third variable regions, conferred binding to additional germline-reverted VRC01 class Abs and activation of germline-reverted BCR of 3BNC60 in transgenic mice following immunization [36, 37]. Deletion of glycan N276 is also one central design feature of engineered outer domain, germline-targeting (eOD-GT) immunogens that bind germline forms of the VRC01 class of bnAbs and activate germline-reverted BCR in knock-in mice [26, 36, 38, 39].

HIV-1 Env is heavily glycosylated, with a glycan content that accounts for approximately 50% of its molecular mass [40]. The majority of these glycans exist as under-processed, high mannose (Man_5_-_9_GlcNac_2_) glycoforms owing to steric constrains imposed by the dense clustering of glycans and the trimerization of gp120-gp41 heterodimers that impede the actions of α-mannosidases required for complex glycan formation [41–44]. A predominance of high mannose glycans is seen with multiple forms of Env produced in different cell types [45–52], where a higher abundance of Man_5_GlcNac_2_ is present on virions and membrane associated Env than on recombinant gp120 and gp140 proteins [41, 45, 48]. The smaller proportion of fully processed glycans exists mainly as sialylated bi-, tri- and tetra-antennary complex-type glycans [4, 48, 53, 54], a portion of which surround the CD4bs [4, 55].

Complex-type glycans are arrested at Man_5_GlcNac_2_ in the absence of the enzyme N-acetylglucosaminyltransferase (GnT1) [56], which is responsible for attachment of GlcNAc to Man_5_GlcNAc_2_ in the medial-Golgi as a requisite step for complete processing. There are reports of improved neutralization potency of mature CD4bs bnAbs against HIV-1 produced in GnT1^-^ cells [27, 57, 58]. Some strains of HIV-1 produced in GnT1^-^ cells are also sensitive to neutralization by germline-reverted forms of V2 apex bnAbs [58]. Here we demonstrate that a combination of Man_5_-enrichment and targeted glycan deletion reduces steric barriers to germline-reverted VRC01-class bnAbs without disrupting functional Env conformation, enabling potent virus neutralization. We describe the implications of these findings for immunogen design and vaccine immune monitoring.

## Results

### Enhanced neutralization potency of mature CD4bs bnAbs against Envs produced in GnT1^-^ cells

Multiple bnAbs were assessed for neutralizing activity against Env-pseudotyped viruses (EPV) produced in either 293T or 293S GnT1^-^ cells. The latter cells were used to generate Man_5_-enriched EPV, with the rationale that relatively small Man_5_ would replace larger complex-type glycans, thereby improving access to the CD4bs. Initially, three mature CD4bs bnAbs (VRC01, 3BNC117 and VRC-CH31) were assayed against EPV expressing Envs from HIV-1 strains CE1176 and WITO. Greater potency (often >10-fold) was seen against GnT1^-^ EPV for all three bnAbs **(Table 1)**.

**Table 1:** GnT1^-^ production enhances the susceptibility of HIV-1 to neutralization by CD4bs bnAbs.

		IC50 (μg/ml)^a^		
EPV^b^	bnAb	293T virus	GnT1^-^ virus	Fold Change
CE1176	VRC01	10.00	0.37	27.0
WITO	VRC01	0.62	0.14	4.4
CE1176	3BNC117	0.63	0.04	15.8
WITO	3BNC117	0.12	0.01	10.9
CE1176	VRC-CH31	3.7	0.30	12.3
WITO	VRC-CH31	0.60	0.01	54.6

^a^Neutralization assays were performed with Env-pseudotyped viruses in TZM-bl cells as described in Materials and Methods.

^b^Env-pseudotyped viruses (EPV) were produced in either HEK 293T or HEK 293s GnT1^-^ cells.

A third EPV, TRO.11, was assayed with a wider range of mature bnAbs covering multiple epitopes **(Table 2)**. With the exception of HJ16 and IgG1b12, the mature CD4bs bnAbs again showed enhanced potency against GnT1^-^ EPV. HJ16 was 32-fold less potent against GnT1^-^ EPV, while IgG1b12 was non-neutralizing. HJ16 requires gp120 glycan N276 [59] and might not tolerate Man_5_GlcNAc_2_ at this site. GnT1^-^ had little or no impact on bnAbs whose epitopes resided outside the CD4bs. Notably, no neutralization was detected with germline-reverted forms of CD4bs bnAbs **(Table 2)**.

**Table 2:** Enhanced susceptibility of GnT1^-^ virus is relatively specific for CD4bs bnAbs.

		IC50 (μg/ml)^a^		
mAb	Epitope	TRO.11/293T^b^	TRO.11/GnT1^-^^b^	Fold change^c^
2G12	Glycan	**0.38**	**0.26**	1.5
2F5	MPER	>50	>50	NA
4E10	MPER	**0.46**	**0.51**	-1.1
10E8	MPER	**0.12**	**0.06**	2.0
DH511.2_K3	MPER	**0.09**	**0.06**	1.5
CH01	V2 apex	>50	>50	NA
PG9	V2 apex	>50	>50	NA
PG16	V2 apex	**1.47**	**1.82**	-1.2
PGDM1400	V2 apex	**0.51**	**0.44**	1.2
PGT121	V3 glycan	**0.04**	**0.09**	-2.3
PGT128	V3 glycan	**0.02**	**0.01**	2.0
10–1074	V3 glycan	**0.01**	**0.02**	-2.0
PGT151	Interface	>50	>50	NA
VRC34.1	Interface	>50	>50	NA
8ANC195	Interface	**0.32**	**0.55**	-1.7
IgG1b12	CD4bs	>50	>50	NA
VRC01	CD4bs	**0.58**	**0.07**	**8.3**
3BNC117	CD4bs	**0.02**	**0.01**	2.0
VRC-CH31	CD4bs	**0.22**	**0.03**	**7.3**
CH103	CD4bs	**6.40**	**0.40**	**16.0**
CH235	CD4bs	**12.20**	**0.09**	**135.6**
3BNC60	CD4bs	**0.06**	**0.02**	**3.0**
HJ16	CD4bs	**0.05**	**1.60**	-**32.0**
sCD4	CD4bs	>50	>50	NA
VRC01gl	CD4bs	>50	>50	NA
VRC-CH31 UCA	CD4bs	>50	>50	NA
CH103 UCA1	CD4bs	>50	>50	NA
CH235 UCA2	CD4bs	>50	>50	NA
3BNC60gl	CD4bs	>50	>50	NA

^a^Neutralization assays were performed with Env-pseudotyped viruses in TZM-bl cells as described in Materials and Methods. Positive values are shown in boldface type.

^b^TRO.11 Env-pseudotyped virus was produced in either HEK 293T or HEK 293s GnT1^-^ cells as indicated.

^c^Changes that are ≥3-fold are shown in boldface type. NA, not applicable (no neutralization detected).

### Complementarity of Man_5_-enrichment and targeted glycan deletion for neutralization by mature CD4bs bnAbs

We next combined GnT1^-^ production and the targeted deletion of one or more glycans surrounding the CD4bs. Mutants of the subtype C transmitted-founder 426c EPV were used that lacked glycan N276 (SM), two glycans at N460 and N463 (DM), or all three glycans (TM1) [27]. A fourth 426c mutant, TM4 (S278R.G471S.N460D.N463D), lacked all three glycans except that glycan N276 was removed by introducing S278R [37]. TM4 also contained a G471S mutation that improves germline VRC01 binding to both eOD-GT6 and 426c [26, 37].

Targeted glycan-deleted EPV, whether produced in 293T or GnT1^-^ cells, maintained a tier 2 neutralization phenotype with HIV-1 sera and were mostly resistant to mAbs that preferentially neutralize tier 1 Envs (non-neutralizing Abs) **(Table 3)**. EPVs produced in GnT1^-^ cells were more sensitive to HIV-1 sera than their 293T-grown counterpart, especially TM1 and TM4, but still within the tier 2 spectrum. These results agree with the tier 2 phenotype of other EPV produced in GnT1^-^ cells [58]. We note that the 293S GnT1^-^ viruses often exhibited lower infectivity than their 293T-grown counterparts. Adjustments were made in the virus inoculum used in the assay to account for this lower infectivity.

**Table 3:** HIV-1 426c variants produced in GnT1^-^ cells retain a tier 2 neutralization phenotype.

		**ID50 (dilution) in TZM-bl**^**a**^				
		**293T Viruses**^**b**^				
**Reagent**	**Epitope**	**426c**	**426c.SM**	**426c.DM**	**426c.TM**	**426c.TM4**
**HIV-1 sera:**						
CHAVI-0293 pool2	Polyclonal	30	30	30	83	177
CHAVI-0537 pool2	Polyclonal	467	182	290	166	323
CHAVI-0642 pool2	Polyclonal	111	64	50	77	86
CHAVI-0461 pool2	Polyclonal	93	56	57	46	102
CHAVI-0598 pool2	Polyclonal	235	150	145	319	476
*Geometric mean titer*		*128*	*78*	*81*	*109*	*189*
**Non-neutralizing Abs:**						
2219	V3	>25	>25	>25	>25	>25
2557	V3	>25	>25	>25	>25	>25
3074	V3	>25	>25	>25	>25	>25
3869	V3	>25	>25	>25	>25	>25
447-52D	V3	>25	>25	>25	>25	>25
838-12D	V3	>25	>25	>25	>25	>25
654-30D	CD4bs	>25	>25	>25	>25	>25
1008-30D	CD4bs	>25	>25	>25	>25	>25
1570D	CD4bs	>25	>25	>25	>25	>25
729-30D	CD4bs	>25	>25	>25	>25	>25
F105	CD4bs	>25	>25	>25	>25	>25
		**293S GnTI**^**-**^ **Viruses**^**b**^				
**Reagent**	**Epitope**	**426c**	**426c.SM**	**426c.DM**	**426c.TM**	**426c.TM4**
**HIV-1 sera:**						
CHAVI-0293 pool2	Polyclonal	45	84	41	299	343
CHAVI-0537 pool2	Polyclonal	109	120	94	111	203
CHAVI-0642 pool2	Polyclonal	206	262	134	258	235
CHAVI-0461 pool2	Polyclonal	66	85	280	56	131
CHAVI-0598 pool2	Polyclonal	566	482	489	897	1452
*Geometric mean titer*		*130*	*161*	*148*	*212*	*315*
**Non-neutralizing Abs:**						
2219	V3	>25	>25	>25	>25	>25
2557	V3	>25	>25	>25	>25	>25
3074	V3	>25	23	>25	>25	>25
3869	V3	>25	>25	>25	>25	>25
447-52D	V3	>25	>25	>25	>25	>25
838-12D	V3	>25	>25	>25	>25	>25
654-30D	CD4bs	>25	>25	>25	>25	>25
1008-30D	CD4bs	>25	>25	>25	>25	>25
1570D	CD4bs	>25	>25	>25	>25	>25
729-30D	CD4bs	>25	>25	>25	>25	>25
F105	CD4bs	>25	>25	>25	>25	>25

^a^Neutralization assays were performed with Env-pseudotyped viruses in TZM-bl cells as described in Materials and Methods.

^b^Env-pseudotyped viruses were produced in either HEK 293T or HEK 293s GnT1^-^ cells. The 426c glycan deletion mutants were SM (N276D), DM (N460D.N463D), TM1 (N276D.N460D.N463D) and TM4 (S278R.G471S.N460D.N463D).

As reported for NIH45-46 [27], mature CD4bs bnAbs were consistently more potent against the 293T version of targeted glycan deleted 426c EPV than against the 293T version of the parental EPV **(Table 4)**. In particular, TM1 and TM4 were 10-1000-fold more susceptible to VRC01 and 3BNC117 compared to parental 426c. DM, TM1 and TM4 were 6-11-fold more sensitive to CH103. TM4 was 4-fold more susceptible to VRC-CH31, whereas no increased in VRC-CH31 susceptibility was seen with DM, and resistance was seen with SM and TM1. Parental and all glycan-deleted 426c EPV produced in 293T cells were resistant to CH235 but were sensitive to CH235.12. Importantly, despite 100-fold and 1000-fold increased potencies of mature VRC01 against the 293T versions of TM1 and TM4, respectively, these EPV were not neutralized by germline-reverted VRC01.

**Table 4:** Neutralization of parental and glycan-modified 426c by mature, intermediate and germline forms of bnAbs.

		IC50 (μg/ml)^a^									
		293T Viruses^b^					293S GnT1^-^ Viruses^b^				
Reagent	Epitope	426c	426c.SM	426c.DM	426c.TM1	426c.TM4	426c	426c.SM	426c.DM	426c.TM1	426c.TM4
**Mature bnAbs:**											
2G12	glycan	>25	>25	>25	>25	>25	>25	>25	>25	>25	>25
2F5	MPER	>25	>25	>25	>25	>25	>25	>25	>25	>25	>25
4E10	MPER	**3.32**	**1.52**	**1.00**	**0.98**	**1.66**	**4**	**3.8**	**3.7**	**4**	**1.6**
10E8	MPER	**0.8**	**1.26**	**1.11**	**1.52**	**0.53**	**0.35**	**0.28**	**0.45**	**0.51**	**0.19**
DH511.2_K3	MPER	**0.8**	**0.77**	**1.50**	**1.06**	**0.56**	**0.85**	**0.87**	**0.75**	**1.2**	**0.56**
CH01	V2 apex	>25	>25	>25	>25	>25	>25	>25	>25	>25	>25
PG9	V2 apex	>5	>5	>5	>5	>5	>5	>5	>5	>5	>5
PG16	V2 apex	>5	>5	>5	>5	>5	>5	>5	>5	>5	>5
PGDM1400	V2 apex	>25	>25	>25	>25	>5	>5	>5	>5	>5	>5
PGT121	V3 glycan	>5	>5	>5	>5	>5	**2.5**	**4.2**	**3.4**	**3.4**	>5
PGT128	V3 glycan	>5	>5	>5	>5	>5	**4.3**	>5	>5	>5	>5
10–1074	V3 glycan	**0.05**	**0.12**	**0.10**	**0.16**	**0.06**	**0.03**	**0.04**	**0.03**	**0.03**	**0.02**
PGT151	gp120/gp41	**0.01**	**0.01**	**0.01**	**0.01**	**0.01**	**1.6**	**1.9**	**2.2**	**2.5**	**2.9**
VRC34.01	gp120/gp41	**0.08**	**0.06**	**0.09**	**0.08**	**0.13**	**0.05**	**0.07**	**0.04**	**0.07**	**0.07**
b12	CD4bs	>25	>25	>25	>25	>25	>25	>25	>25	>25	>25
HJ16	CD4bs	>25	>25	>25	>25	>25	>25	>25	>25	>25	>25
3BNC117	CD4bs	**0.20**	**0.24**	**0.13**	**0.01**	**0.003**	**0.02**	**0.01**	**0.006**	**0.003**	**<0.001**
VRC-CH31	CD4bs	**0.62**	>25	**0.73**	>25	**0.15**	**0.04**	**0.7**	**0.02**	**1.6**	**0.01**
CH103	CD4bs	>40	>40	**6.1**	**5.2**	**3.7**	**5.3**	**2.2**	**0.63**	**0.09**	**0.48**
CH235	CD4bs	>50	>50	>50	>50	>25	>25	>25	>25	>25	>25
CH235.12	CD4bs	**8.95**	**1.08**	**0.66**	**0.09**	**0.1**	>25	**0.26**	>25	**0.07**	**24.2**
VRC01	CD4bs	**2.20**	**0.39**	**0.41**	**0.03**	**0.002**	**0.19**	**0.04**	**0.04**	**0.015**	**0.007**
VRC03	CD4bs	nt^c^	nt	nt	nt	**0.003**	**0.014**	**0.003**	**0.005**	**0.003**	**<0.002**
VRC04	CD4bs	nt	nt	nt	nt	**0.013**	**0.18**	**0.02**	**0.05**	**0.01**	**<0.002**
VRC07	CD4bs	nt	nt	nt	nt	**<0.002**	**0.07**	**0.02**	**0.05**	**0.009**	**<0.002**
VRC18b	CD4bs	nt	nt	nt	nt	**0.005**	**0.06**	**0.01**	**0.02**	**0.006**	**<0.002**
VRC20 (VRC-PG20)	CD4bs	nt	nt	nt	nt	**<0.002**	**0.015**	**0.004**	**0.007**	**0.005**	**0.008**
VRC23	CD4bs	nt	nt	nt	nt	**0.082**	**0.19**	**0.1**	**0.06**	**3.1**	**<0.002**
12A12	CD4bs	nt	nt	nt	nt	**0.003**	**0.06**	**0.01**	**0.01**	**0.01**	**<0.002**
**UCAs and intermediate Abs:**											
VRC01gl	CD4bs	>50	>50	>50	>50	>25	>50	**0.99**	>50	**1.37**	**0.36**
VRC03gl	CD4bs	nt	nt	nt	nt	>25	>25	>25	>25	>25	>25
VRC04gl	CD4bs	nt	nt	nt	nt	>25	>25	>25	>25	>25	>25
VRC07gl	CD4bs	nt	nt	nt	nt	>25	>25	**0.76**	>25	**1.6**	**1.7**
VRC18bgl	CD4bs	nt	nt	nt	nt	>25	>25	>25	>25	**23**	**17.3**
VRC20gl	CD4bs	nt	nt	nt	nt	**2.39**	>25	**10.3**	>25	**4.6**	**0.03**
+VRC23gl	CD4bs	nt	nt	nt	nt	>25	>25	>25	>25	>25	>25
12A12gl	CD4bs	nt	nt	nt	nt	>25	>25	>25	>25	>25	**0.63**
3BNC117gl	CD4bs	nt	nt	nt	nt	>25	>25	>25	>25	>25	>25
VRC-CH31_UCA1	CD4bs	>50	nt	>50	nt	>25	nt	nt	>50	nt	>25
VRC-CH31_I4	CD4bs	**1.32**	nt	**0.59**	nt	**0.02**	nt	nt	**0.018**	nt	**<0.011**
VRC-CH31_I3	CD4bs	**1.39**	nt	**0.84**	nt	**0.01**	nt	nt	**0.021**	nt	**<0.011**
VRC-CH31_I2	CD4bs	**1.52**	nt	**0.89**	nt	**0.07**	nt	nt	**0.02**	nt	**<0.011**
VRC-CH31_I1	CD4bs	**1.94**	nt	**1.15**	nt	**0.15**	nt	nt	**0.032**	nt	**<0.011**
CH103_UCA1.1_4A	CD4bs	>50	nt	nt	>50	>25	nt	nt	nt	>50	>25
CH103_UCAGrand5	CD4bs	>50	nt	nt	>50	>25	nt	nt	nt	>50	>25
CH103_IA_9_4A	CD4bs	>50	nt	nt	>50	>25	nt	nt	nt	>50	>25
CH103_IA_8_4A	CD4bs	>50	nt	nt	>50	>25	nt	nt	nt	>50	>25
CH103_IA_7_4A	CD4bs	>50	nt	nt	>50	>25	nt	nt	nt	>50	>25
CH103_IA_6_4A	CD4bs	>50	nt	nt	>50	>25	nt	nt	nt	>50	>25
CH103_IA_5_4A	CD4bs	>20	nt	nt	>20	>25	nt	nt	nt	>20	>20
CH103_IA_4_4A	CD4bs	>50	nt	nt	>50	>25	nt	nt	nt	>50	>25
CH235 UCA2	CD4bs	>50	nt	nt	>50	>50	nt	nt	nt	>50	>50
CH235_I4_v2_4A	CD4bs	>50	nt	nt	>50	>25	nt	nt	nt	>50	>25
CH235_I3_v2_4A	CD4bs	>50	nt	nt	>50	>25	nt	nt	nt	>50	>25
CH235VH_I1_v2_4A	CD4bs	>50	nt	nt	>50	>25	nt	nt	nt	>50	>25

^a^Neutralization assays were performed with Env-pseudotyped viruses in TZM-bl cells as described in Materials and Methods. Light shade, 0.5–25 μg/ml; middle shade, 0.01–0.49 μg/ml; dark shade, <0.01 μg/ml.

^b^Env-pseudotyped viruses were produced in either HEK 293T or HEK 293s GnT1^-^ cells. The 426c glycan deletion mutants were SM (N276D), DM (N460D.N463D), TM1 (N276D.N460D.N463D) and TM4 (S278R.G471S.N460D.N463D).

^c^nt, not tested.

**Fig 1:**
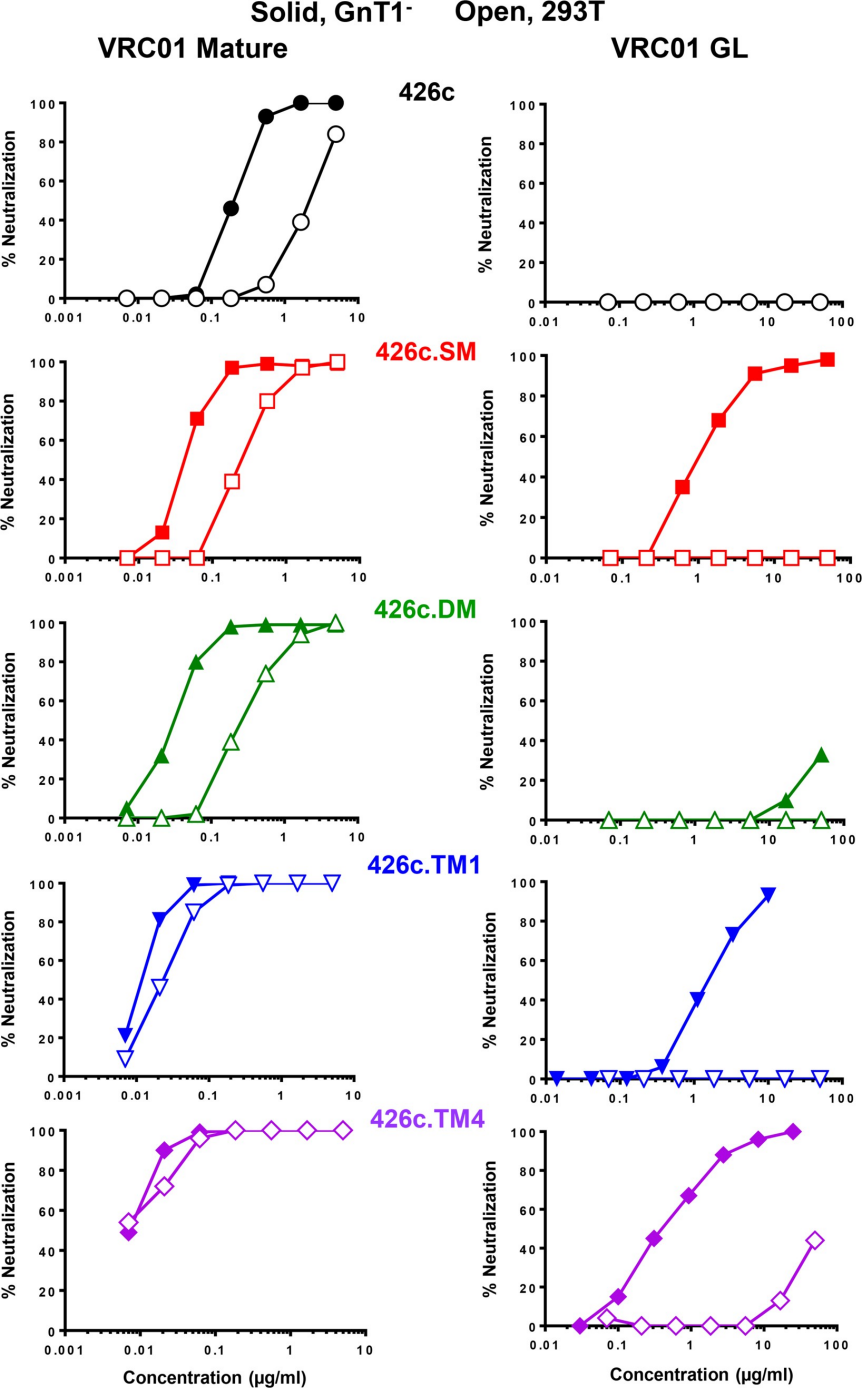
Complementarity of targeted glycan-deletion and Man_5_-enrichement for neutralization by germline-reverted VRC01. Parental and glycan deletion mutants of 426c were produced as Env-pseudotyped viruses in 293T and 293S GnT1^**-**^ cells and assayed for neutralization by mature and germline-reverted VRC01 in TZM-bl cells. The 426c glycan deletion mutants were SM (N276D), DM (N460D.N463D), TM1 (N276D.N460D.N463D) and TM4 (S278R.G471S.N460D.N463D).

GnT1^-^ production increased the sensitivity of 426c by 8–12 fold when assayed with mature VRC01, 3BNC117, VRC-CH31 and CH103, and this sensitivity increased further by targeted glycan deletion, thereby demonstrating the complementary nature of GnT1^-^ production and targeted glycan deletion for enhanced neutralization by these mature bnAbs **(Table 4)**. Notably, GnT1^-^ increased the sensitivity of SM and DM to mature VRC01 by ~10-fold. A further increase in sensitivity was seen against TM1 and TM4 but here GnT1^-^ provided little or no benefit **(Table 4 and Fig 1)**, indicating that mature VRC01 does not require Man_5_-enrichment for maximum neutralization of these triple glycan-deleted viruses. GnT1^-^ increased the sensitivity of all four 426c glycan mutants to neutralization by 3BNC117 and, to a lesser extent, by VRC-CH31 **(Table 4)**. In contrast, GnT1^-^ rendered parental 426c, DM and TM4 less sensitive to CH235.12. GnT1^-^ had little impact on most other mature bnAbs tested. A notable exception was a ~100-fold diminished potency of the PGT151 (gp120/gp41 interface bnAb) against GnT1^-^ versions of all four glycan mutants of 426c, which has been observed for other viruses as well [58] and is consistent with the known dependency of this bnAb on complex-type glycans [60, 61]. GnT1^-^ had no measurable impact on VRC34.01, whose epitope overlaps but is distinct from that of PGT151 [62].

### Neutralization by germline-reverted forms of VRC01-class bnAbs requires a combination of Man_5_-enrichment and glycan deletion

The elevated sensitivity of GnT1^-^ versions of glycan-deleted 426c EPV to neutralization by certain mature CD4bs bnAbs led us to test whether the viruses would be sensitive to germline-reverted and early intermediates of CD4bs bnAbs. These tests included near-germline forms of several VRC01-class bnAbs in addition to fully reverted germline forms of CH103, CH235 and CH235.12. Mature CH235 and CH235.12 are members of the same lineage and exhibit 18% and 90% neutralization breadth, respectively, against a multiclade panel of 199 viruses [2]. Their unmutated common ancestor (UCA) is referred to here as CH235 UCA2.

GnT1^-^ versions of the SM, TM1 and TM4 were remarkably sensitive to neutralization by germline-reverted VRC01, with IC50s of 0.99, 1.37 and 0.36 μg/ml, respectively **(Table 4 and Figs 1 and S1)**. DM, which retains glycan 276, was the only mutant not neutralized. VRC01 has been shown to bind gp140 trimers of SM and TM1 but not DM [27], leading to the suggestion that germline VRC01 recognizes viruses lacking glycan N276 and that accommodating this glycan leads to breadth [63]. Our results agree with this interpretation and suggest that Man_5_ enrichment will further improve germline binding to functional Env trimers.

293T versions of the glycan-deleted 426c EPVs were not susceptible to neutralization by germline-reverted VRC01, although we note a minor positive deflection against the 293T version of TM4 **(Fig 1)**. Parental 426c resisted neutralization regardless of the cells used for EPV production **(Fig 1)**. Thus, germline-reverted VRC01 neutralizes 426c when the Env is both Man_5_-enriched and lacking glycan N276.

**Fig 2:**
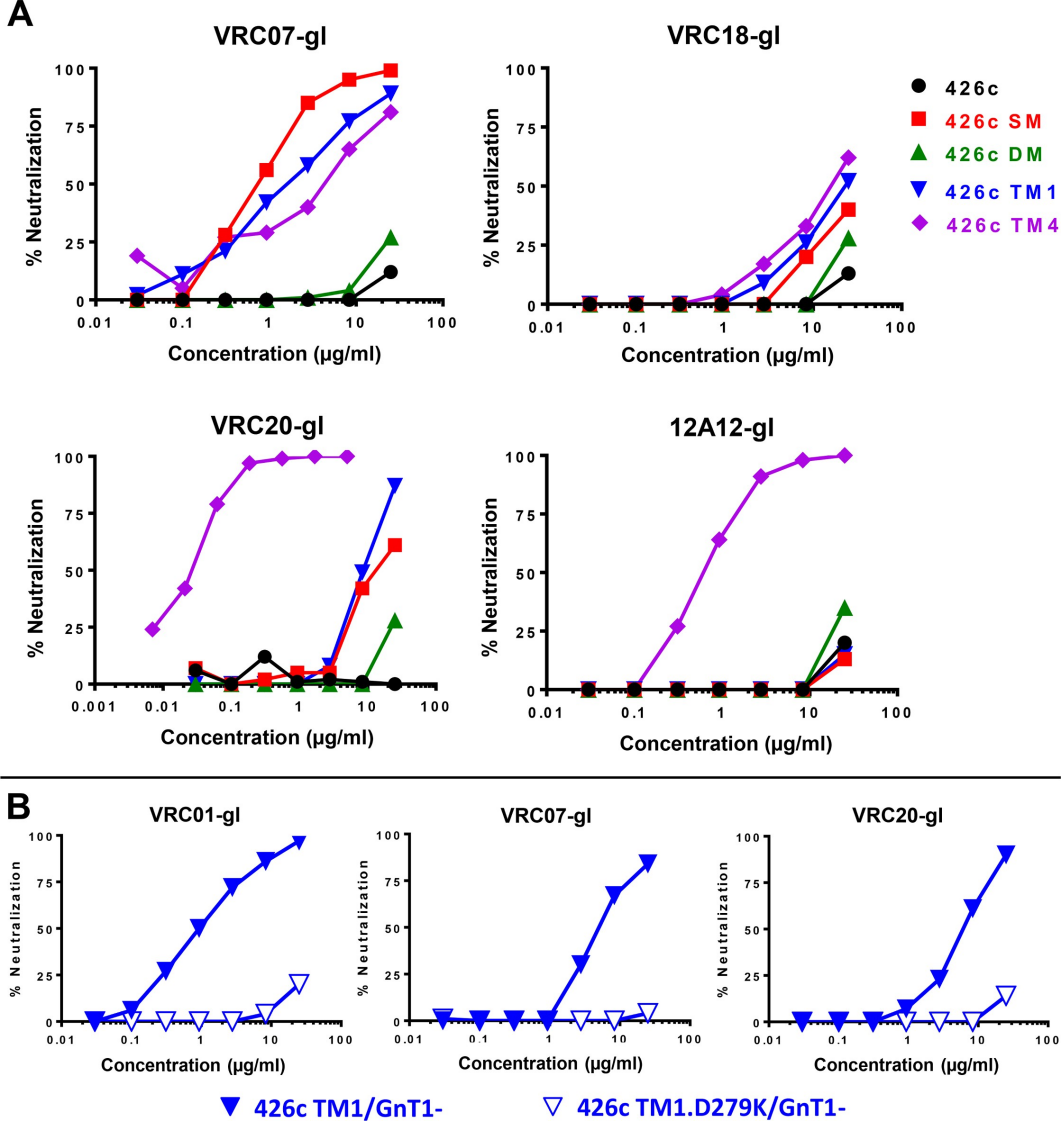
Detection and epitope mapping of neutralization by germline-reverted forms of VRC01-class bnAbs. (A) Germline reverted forms of the indicated bnAbs were assayed in TZM-bl cells against Env-pseudotyped viruses 426c, 426c.SM (N276D), 426c.DM (N260D.N463D), 426c.TM1 (N276D.N460D.N463D) and 426c.TM4 (S278R.G471S.N460D.N463D) produced in 293S GnT1^**-**^ cells. (B) Germline-reverted forms of VRC01, VRC07 and VRC20 were assayed in TZM-bl cells against 426c.TM1 and 426c.TM1.D279K produced in 293S GnT1^**-**^ cells.

GnT1^-^ versions of SM, TM1 and TM4 were also susceptible to neutralization by germline forms of other VRC01-class bnAbs **(Table 4 and Fig 2A)**. SM, TM1 and TM4 were sensitive to VRC07gl and VRC20gl. TM1 was in addition sensitive to VRC18gl, while TM4 was in addition sensitive to VRC18gl and 12A12gl. No neutralization was detected with germline forms of VRC03, VRC04, VRC23, 3BNC117 and VRC-CH31 **(Table 4)**. Thus, GnT1^-^ versions of SM, TM1 and TM4 are highly susceptible to neutralization by some but not all germline-reverted forms of VRC01-class bnAbs. SM was the most sensitive to VRC07gl (0.76 μg/ml), whereas TM4 was the most sensitive to VRC18gl (17.3 μg/ml), VRC20gl (0.03 μg/ml) and 12A12gl (0.63 μg/ml).

The ability to detect germline-reverted forms of certain VRC01-class bnAbs in neutralization assays suggests utility for monitoring early precursors in clinical trials. Neutralization-based evidence that such precursors exist in serum samples would be strengthened by confirming VRC01-like epitope specificity. To enable epitope mapping, we introduced a known VRC01 resistance mutation, D279K [63], into TM1 and show that the GnT1^-^ version of this mutant is highly resistant to germline forms of VRC01, VRC07 and VRC20 (**Fig 2B**).

In addition to germline-reverted bnAbs, we also assessed intermediate forms of VRC-CH31, CH103 and CH235. Due to limited quantities, the intermediates of CH103 and CH235 were assayed only with the TM1 and TM4, which were the most susceptible to germline forms of VRC01-class bnAbs. As shown in **Table 4**, these EPVs were resistant to the intermediate antibodies regardless of whether the EPVs were produced in 293T or GnT1^-^ cells. Assays with intermediates of VRC-CH31 were performed with DM and TM4 as the two mutants that were most sensitive to the mature bnAb. 239T versions of both mutants were sensitive to all four VRC-CH31 intermediates, with DM being slightly more sensitive than parental 426c. Far greater potency (>10-fold) was seen when DM was produced in GnT1^-^ cells, suggesting that the GnT1^-^ version of this Env may provide an advantage for engaging and detecting early intermediates of the VRC-CH31 lineage.

As an initial test of utility for immunogen design, the binding affinity of germline-reverted VRC01 was measured against 293T and GnT1^-^ versions of uncleaved gp140s from SM and TM1. Strong (nM apparent affinity) binding was detected in all cases and was stronger against TM1 than SM gp140; however, no improvement was seen with GnT1^-^ version of the gp140s **(Table 5)**. These results indicate that GnT1^-^ provides no germline-targeting advantage for uncleaved gp140. Unfortunately, GnT1^-^ versions of corresponding SOSIPs, which exhibit a more native-like structure, were not available.

**Table 5:** Germline-reverted VRC01 Fab binding to 426c Env variants produced in 293T and GnT1^-^ cells.

Env (producer line)	KD (M)	kon(1/Ms)	kon Error	koff(1/s)	koff Error
426c N276D gp140 (293T)	5.88E-08	9.01E+03	3.27E+02	1.93E-04	2.50E-05
426c N276D gp140 (GnT1^-^)	1.78E-07	1.11E+04	3.09E+02	7.39E-04	1.89E-05
TM1 gp140 (293T)	2.94E-08	5.03E+03	6.97E+01	1.48E-04	3.23E-06
TM1 gp140 (GnT1^-^)	4.26E-08	5.37E+03	8.43E+01	2.29E-04	2.74E-06

## Discussion

Most current HIV-1 vaccine candidates are unable to engage appropriate germline B cells that give rise to bnAbs. To overcome this obstacle, researchers are identifying natural and engineered Env proteins that bind germline-reverted forms of the bnAbs as partial mimics of the naïve B cell receptors [2, 6, 26, 27, 37–39, 64–66]; such proteins are in early stages of development and it is unclear whether they will initiate correct antibody lineages in humans and/or wild-type animal models. We sought Env glycan modifications that would permit germline forms of CD4bs bnAbs to neutralize EPV as stringent proof of functional Env trimer binding. A previous study demonstrated weak neutralization by germline-reverted CH103 against an early autologous tier 1 EPV [30]. Another study demonstrated neutralization by germline-reverted NIH45-46 (a clonal variant of VRC01) against 293T versions of SM and TM1 but only at very high antibody concentrations (IC50 ~100 μg/ml) [27]. We identified a combination of Env glycan modifications that permit far greater neutralization potency by near germline forms of multiple VRC01-class bnAbs.

Man_5_-enrichment in GnT1^-^ cells was hypothesized to reduce steric barriers to germline bnAb binding without disrupting native Env conformation. That Man_5_-enriched Envs were infectious and maintained a tier 2 neutralization phenotype is consistent with previous reports [46, 55, 58] and indicates that native conformation was indeed preserved, at least in large part. Several mature CD4bs bnAbs were substantially more potent against Man_5_-enriched Envs than wild type Envs, while most bnAbs to epitopes outside the CD4bs were unaffected. One exception is the negative impact Man_5_-enrichment had on PGT151, which was observed before [58] and agrees with previous reports that PGT151 requires one or more complex-type glycans [60, 61]. In another study, Man_5_-enrichment had only modest effects on mature CD4bs bnAbs and had more dramatic effects on V2 apex and gp120/gp41 interface bnAbs than we observed [58]. Because of the many strain specific effects observed [58], discrepancies between the two studies are likely explained by the different viruses used.

A combination of Man_5_-enrichment and targeted glycan deletion of 426c EPV was required for neutralization by germline-reverted forms of VRC01-class bnAbs. A simple explanation for why both modifications were necessary is that targeted glycan deletion alone did not remove all complex-type glycans that serve as steric barriers to germline binding. Indeed, reducing the glycan density on Env by targeted-glycan deletion has potential to relieve steric constraints on α-mannosidases and result in an increased number of remaining glycans that are fully processed [41–44]. Any additional complex-type glycans generated in this way should remain arrested as smaller Man_5_ glycoforms when produced in GnT1^-^ cells, thereby affording a lower barrier to germline binding without the unwanted consequence of creating additional complex-type glycans.

Our observations raise the possibility that heterogeneity in Env sequon location and occupation, and in the composition of glycans at occupied sites [50, 52, 53] contribute to VRC01-class bnAb responses in HIV-1 infected individuals, where part of the maturation process appears to involve an ability to accommodate glycan N276 and surrounding complex-type glycans. Our observations also suggest that Man_5_-enrichment has potential to improve germline-targeting to initiate VRC01-class bnAbs, especially when combined with other germline-targeting design features [27, 37, 39]. How well these glycan modifications will perform with native versus non-native forms of Env is uncertain; however, it is important to emphasize that our findings reflect antibody interactions with functional Env trimers as the sole targets for neutralization. This might explain why GnT1^-^ provided no advantage for germline-reverted VRC01 binding to uncleaved gp140 trimers. A more native form of the trimer might be necessary to gain the benefit of Man_5_-enrichment. Also, because the germline-reverted VRC01-class bnAbs used here and in previous studies contain mature HCDR3 and J regions, additional 426c Env modifications might be needed to initiate the earliest stages of this bnAb class.

Another application of our findings is the detection of early precursors of VRC01-like bnAbs in vaccine studies. Detection of early precursors in a high throughput neutralization assay would complement other technologies, such as antigen-specific memory B cell sorting and immunoglobulin sequence analyses. Based on relative sensitivities, Man_5_-enriched SM is optimal for detecting VRC07gl, while Man_5_-enriched TM4 is optimal for VRC01gl, VRC18gl, VRC20gl and 12A12gl. Both viruses should be useful to screen for the presence of vaccine-elicited VRC01-like bnAb precursors. Addition evidence for the presence of these precursors is obtainable by showing whether the activity is abrogated by the D279K resistance mutation. Thus, a complete signature for VRC01-like precursors is the ability to neutralize 426c in a manner that is dependent on both glycan-deletion and Man_5_-enrichment, and is abrogated by D279K. Until the technology is refined to capture a wider range of VRC01 class precursors, negative neutralization should not be interpreted as evidence that precursors are absent. In cases where a positive signature is detected, additional confirmation of the presence of VRC01-like precursors should be sought at the molecular level. Indeed, the signature should prove useful as an initial screening tool to identify interesting cases for deeper interrogation.

In summary, converting complex-type glycans to smaller Man_5_ glycoforms on 426c Env complements targeted glycan deletion strategies in reducing steric barriers to near germline forms of VRC01 without disrupting functional Env conformation. This finding is based on virus neutralization as a specific measure of functional Env trimer binding and suggests application to vaccines that aim to elicit VRC01-like bnAbs. Preservation of the fusion-competent structure of glycan-modified 426c Env might be essential for early B cell interactions that initiate the bnAb lineage. Finally, a set of virus reagents identified here should be useful for monitoring the presence of early VRC01-like precursors in vaccine trials.

## Methods

### Ethics statement

This study utilized pre-existing, de-identified human serum samples under approval of the Duke University Health System Institutional Review Board (Pro00015593). The data were analyzed anonymously.

### Cells

TZM-bl, HEK 293T/17 and HEK 293S/GnT1^-^ cells were maintained in Dulbecco's Modified Eagle's Medium (DMEM) containing 10% fetal bovine serum (FBS) and gentamicin (50 μg/ml) in vented T-75 culture flasks (Corning-Costar). Cultures were incubated at 37°C in a humidified 5% CO2–95% air environment. Cell monolayers were split 1:10 at confluence by treatment with 0.25% trypsin, 1 mM EDTA.

### Antibodies and HIV-1 sera

The monoclonal antibodies used here were described previously: CD4bs bnAbs VRC01, VRC03, VRC04, VRC07, VRC-18b, VRC20, VRC23, 12A12 [8–10, 24], 3BNC117, 3BNC60 [7], VRC-CH31 [67], N6 [5], HJ16 [3] and IgG1b12 [68]; high mannose glycan-specific bnAb 2G12 [69]; gp41 membrane proximal external region (MPER)-specific bnAbs 2F5, 4E10 [70], 10E8 [71] and DH511.2_K3 [72]; V2 apex bnAbs PG9, PG16 [73], CH01 [67] and PGDM1400 [74]; V3 glycan bnAbs PGT121, PGT128 and 10–1074 [57, 75]; gp41-gp120 interface bnAbs PGT151 [60] and VRC34.01 [62]. VRC01, VRC34.01 and 10E8 were produced by the Vaccine Research Center, NIH. N6 was obtained from Dr. Mark Connors. 3BNC117, 3BNC60 and 10–1074 were obtained from Dr. Michel Nussenzweig. VRC-CH31 and CH01 were produced by Catalent Biologics (Madison, WI). DH511.2_K3 was produced by the Human Vaccine Institute, Duke University Medical Center. HJ16 was obtained from Dr. Davide Corti. IgG1b12, 2G12, 2F5, 4E10, PG9 and PG16 were purchased from Polymun Scientific (Klosterneuburg, Austria). PGDM1400, PGT121, PGT128 and PGT151 were a kind gift from Dr. Dennis Burton. VRC01, VRC03, VRC04, VRC07, VRC-18b, VRC20, VRC23, 12A12, 3BNC117, 3BNC60, VRC-CH31 and N6 belong to the VRC01-class of bnAbs characterized by heavy-chain mimicry of the CD4 receptor, VH1-2 germline gene usage, and a 5-amino acid CDRL3. VRC01, VRC03 and VRC07 are clonally related.

In addition to these mature bnAbs, we utilized UCAs, intermediates and mature forms of CH103, CH235/CH235.12 [2, 30] and VRC-CH31 [10], which were produced by the Human Vaccine Institute, Duke University Medical Center, Durham, North Carolina. The unmutated common ancestor (UCA) sequence for the CH235/CH235.12 lineage used in this study differs by one amino acid from the UCA described previously [2]. The UCA used here, which we refer to as CH235 UCA2, has a methionine in the 4th position of the light chain in place of a leucine in the previously described UCA version. Other antibodies included germline-reverted forms of the VRC01-class bnAbs VRC01, VRC03, VRC04, VRC07, VRC18b, VRC20, VRC23, 12A12 and 3BNC117 [9, 10, 24, 27], which were produced at the Vaccine Research Center, NIH. These latter germline-reverted antibodies possess mature HCDR3 and J regions whose germlines could not be inferred with existing sequence information. Sequences of these germline-reverted antibodies are shown in S1 Table.

Neutralization tier phenotyping was performed with serum pools from individuals in southern Africa (South Africa, Malawi and Tanzania) who participated in a CHAVI study of chronic HIV-1 infection (CHAVI samples 0406, 0060, 0642, 0293, 0598, 0537, 0468, 0461, 0382 and 0134). These study subjects had all been infected for at least three years. Samples from 6–10 time points collected over 8–60 months were pooled on a per-subject basis and heat-inactivated for 30 minutes at 56°C. For deeper interrogation of neutralization phenotype, a set of monoclonal antibodies that show a strong preference for tier 1 viruses was used. This set included V3-specific antibodies 2219, 2557, 3074, 3869, 447-52D and 838-D, and the CD4bs antibodies 654-30D, 1008-30D, 1570D, 729-30D and F105, all produced by Drs. Susan Zolla-Pazner and Miroslaw K. Gorny at New York University and the Veterans Affairs Medical Center, New York, New York.

### Pseudotyping Envs

Full-length functional HIV-1 Envs were used for virus pseudotyping. Previous reports described Envs for strains CE1176 [76], WITO [77], TRO.11 [77], CH0505TF and CH0505.w4.3 [2]. Glycan deleted Envs CH0505TF.gly4, CH0505TF.gly197, CH0505TF.gly3.276 and CH0505TF.gly3.461 were described by Zhou *et al*. [49, 78]. Envs for 426c and the glycan deleted variants SM (N276D), DM (N460D.N463D), TM1 (N276D.N460D.N463D) and TM4 (S278R.G471S.N460D.N463D) were described by McGuire *et al*. [27, 37]. In some cases additional mutations introduced by site-directed mutagenesis as described [79].

### Transfection

Env-pseudotyped viruses were produced in either 293T/17 or 293S GnT1^-^ cells (American Type Culture Collection) as described [80]. 293S GnT1^-^ cells lack the enzyme N-acetylglucosaminyltransferase and have been shown to yield HIV-1 Envs that contain Man_6-9_ glycoforms and are enriched for under-processed Man_5_ glycoforms in place of complex glycans [46, 55]. Env-pseudotyped viruses were generated by transfecting exponentially dividing 293T/17 or 293S GnT1^-^ cells (5 X 10^6^ cells in 12 ml growth medium in a T-75 culture flask) with 4 μg of rev/env expression plasmid and 8 μg of an env-deficient HIV-1 backbone vector (pSG3ΔEnv), using Fugene 6 transfection reagent. Cells were washed after 3–8 hours and incubated in fresh growth medium without transfection reagents. Env-pseudotyped virus-containing culture supernatants were harvested 2 days after transfection, filtered (0.45 μm), and stored at -80°C in 1 ml aliquots. Infectivity was quantified in TZM-bl cells by performing serial fivefold dilutions of pseudovirus in quadruplicate wells in 96-well culture plates in a total volume of 100 μl of growth medium for a total of 11 dilution steps. Freshly trypsinized cells (10,000 cells in 100 μl of growth medium containing 75 μg/ml DEAE-dextran) were added to each well, and the plates were incubated at 37°C in a humidified 5% CO_2_−95% air environment. After a 48-hour incubation, 100 μl of culture medium was removed from each well and 100 μl of Britelite reagent was added to the cells. After a 2-min incubation at room temperature to allow cell lysis, 150 μl of cell lysate was transferred to 96-well black solid plates (Corning-Costar) for measurements of luminescence using a Victor 3 luminometer (Perkin-Elmer Life Sciences, Shelton, CT). A dilution of virus that results in 50,000–250,000 relative luminescence units (RLUs) was used for neutralization assays.

### Neutralization assay

Neutralization assays were performed in TZM-bl cells (NIH AIDS Research and Reference Reagent Program, contributed by John Kappes and Xiaoyun Wu) as described [80]. Briefly, a pre-titrated dose of Env-pseudotyped virus was incubated with serial 3-fold or 5-fold dilutions of test sample in duplicate in a total volume of 150 μl for 1 hr at 37°C in 96-well flat-bottom culture plates. Freshly trypsinized cells (10,000 cells in 100 μl of growth medium containing 20 μg/ml DEAE dextran) were added to each well. One set of control wells received cells + virus (virus control) and another set received cells only (background control). After 48 hours of incubation, the cells were lysed by the addition of Britelite (PerkinElmer Life Sciences) and three quarters of the cell lysate was transferred to a 96-well black solid plate (Costar) for measurement of luminescence. Neutralization titers are either the serum dilution (ID50) or antibody concentration (IC50) at which relative luminescence units (RLU) were reduced by 50% compared to virus control wells after subtraction of background RLUs. For all reported results, the average RLU of virus control wells was >10 times the average RLU of cell control wells, the percent coefficient of variation (%CV) between RLU in the virus control wells was ≤30%, the percent difference between duplicate wells was ≤30%, and neutralization curves cross the 50% neutralization cut-off 0–1 times. Unless otherwise stated, the values and neutralization curves in the manuscript are from a single assay in (duplicate wells) that passed these quality control criteria.

### Soluble recombinant Env gp140 trimers

Plasmids encoding recombinant His-tagged SM and TM1 uncleaved 426c gp140 trimers were expressed by transient transfection in HEK 293T or HEK 293S (GnT1^-^) cells as described previously [37]. Secreted envelope proteins were purified from conditioned media using Ni-NTA resin (Qiagen) followed by 16/60 S200 size-exclusion chromatography into PBS buffer. SEC fractions containing trimeric gp140 were pooled, aliquoted, frozen in liquid nitrogen and stored at -20°C until further use.

### Biolayer interferometry

Kinetic analysis of antibody-binding to 426c uncleaved soluble gp140 trimers was performed by BLI as described previously [81] using the Octet Red instrument (ForteBio, Inc, Menlo Park, CA) at 29°C with shaking at 500 r.p.m. Briefly, germline-reverted VRC01 IgG was immobilized on anti-AHC biosensors (40 μg/ml in PBS) for 240s. Sensors were then incubated for 1 min in kinetic buffer (KB: 1X PBS pH7.4, 0.1% BSA, 0.02% Tween 20 and 0.05% NaN_3_) to establish the baseline signal. Antibody loaded sensors were then immersed into 2-fold dilution series of Env trimers in KB (ranging from 4 μM to >250 nM) for 300 seconds association, followed by immersion in KB alone for 600 seconds dissociation. Measurements of Env-Ab binding were corrected by subtracting the background signal obtained from duplicate Env traces generated with an Env-irrelevant control IgG. Curve fitting to determine relative apparent antibody affinities for envelope was performed using a 1:1 binding model and ForteBio data analysis software. Mean k_on_, k_off_, and K_D_ values were determined by averaging all the binding curves within a dilution series (all R^2^ values greater than 95% confidence level).

## Supporting information

S1 Fig Repeat assay results for VRC01gl. **Shown are results for VRC01gl assayed against GnT1- versions of 426c.TM1 (n = 5) and 426c.TM4 (n = 4).** Due to a limited supply of the antibody, some concentration ranges are lower than others. Accompanies Fig 1. Fig 1 shows the curves for assay 3 for both viruses as representing the closest to the mean.
(PDF)

S1 Table Amino acid sequences of germline-reverted antibodies. (XLSX)
