# Correction: HIV-1 envelope glycan modifications that permit neutralization by germline-reverted VRC01-class broadly neutralizing antibodies

**DOI:** 10.1371/journal.ppat.1007646

**Published:** 2019-03-26

**Authors:** Celia C. LaBranche, Andrew T. McGuire, Matthew D. Gray, Shay Behrens, Xuejun Chen, Tongqing Zhou, Quentin J. Sattentau, James Peacock, Amanda Eaton, Kelli Greene, Hongmei Gao, Haili Tang, Lautaro G. Perez, Kevin O. Saunders, Peter D. Kwong, John R. Mascola, Barton F. Haynes, Leonidas Stamatatos, David C. Montefiori

Peter D. Kwong and Xuejun Chen are not included in the author byline. Xuejun Chen should be listed as the fifth author and Peter D. Kwong should be listed as the fifteenth author. Both are affiliated with Vaccine Research Center, National Institute of Allergy and Infectious Diseases, National Institutes of Health, Bethesda, Maryland, USA. The contributions of these authors are as follows: Investigation, Resources.
