# Supplementary material for: HIV-1 envelope glycan modifications that permit neutralization by germline-reverted VRC01-class broadly neutralizing antibodies
Source: PLoS Pathog. 2018 Nov 5;14(11):e1007431. doi: 10.1371/journal.ppat.1007431 (PMC6237427; doi:10.1371/journal.ppat.1007431)
Supplement: S1 Fig — Shown are results for VRC01gl assayed against GnT1- versions of 426c.TM1 (n = 5) and 426c.TM4 (n = 4). Due to a limited supply of the antibody, some concentration ranges are lower than others. Accompanies Fig 1. Fig 1 shows the curves for assay 3 for both viruses as representing the closest to the mean. (PDF) [file ppat.1007431.s001.pdf]

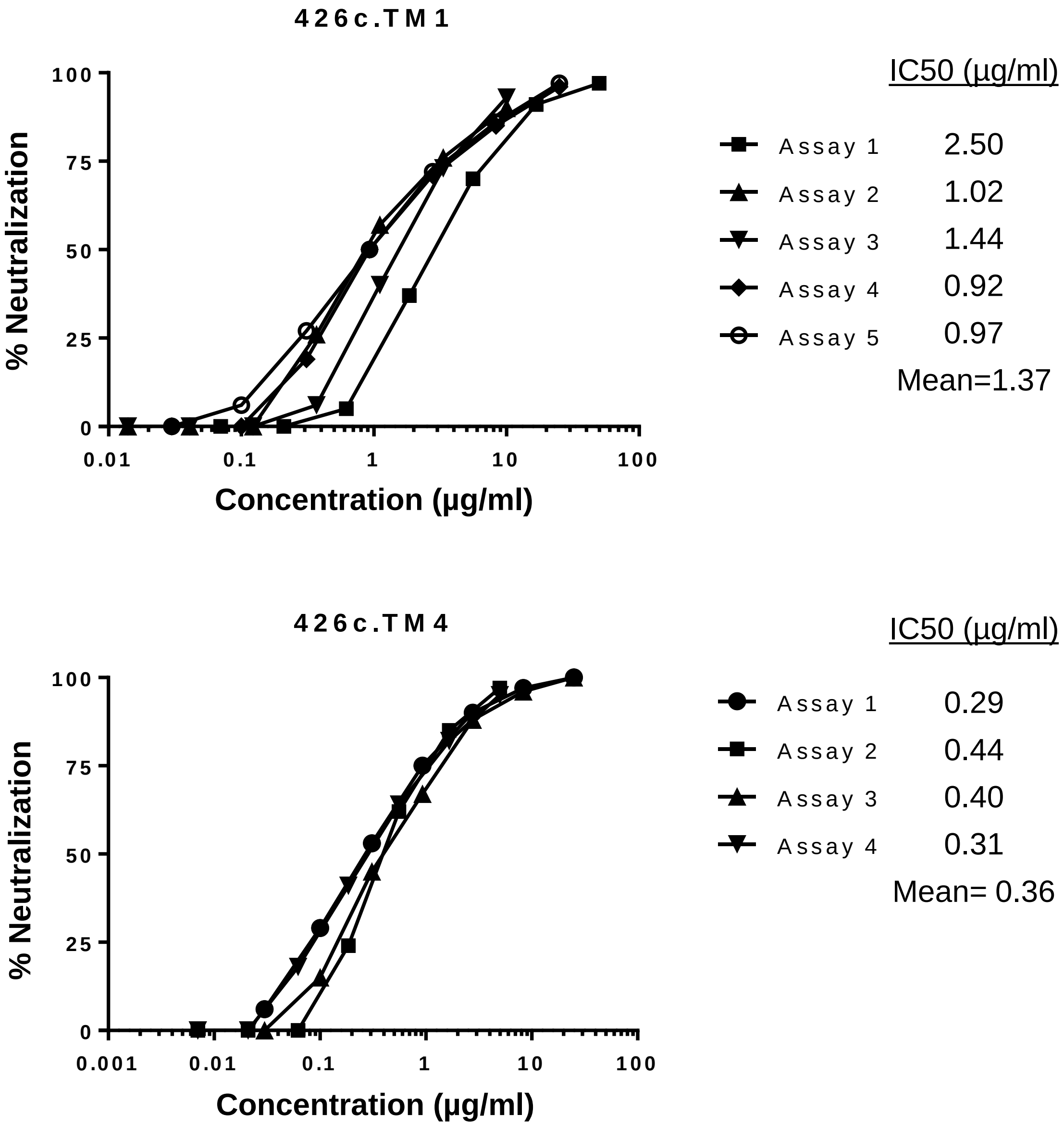

S1 Fig. Repeat assay results for VRC01gl. Shown are results for VRC01gl assayed against $GnT1^-$ versions of 426c.TM1 (n=5) and 426c.TM4 (n=4). Due to a limited supply of the antibody, some concentration ranges are lower than others. Accompanies Fig 1. Figure 1 shows the curves for assay 3 for both viruses as representing the closest to the mean.
